# Temperature Changes of NaOCl after Irrigation Using Passive Ultrasonic Irrigation, Easy Clean, and XP-Endo Finisher: A Randomized Crossover Clinical Trial

**DOI:** 10.1055/s-0044-1791685

**Published:** 2025-03-12

**Authors:** Geraldo Edson Freitas Athayde de Moraes, Daniel Guimarães Pedro Rocha, Carlos Eduardo Fontana, Rina Andréa Pelegrine, Alexandre Sigrist de Martin, Índia Olinta De Azevedo Queiroz, Carlos Eduardo da Silveira Bueno

**Affiliations:** 1 Conservative surgery Department, Collage of Oral and Dental Surgery, Misr University for Science and Technology (MUST), Giza, Egypt; 2 Oral Pathology department, Faculty of Dentistry, Sinai University, Kantara Campus, Ismailia, Egypt; 3 Pediatric Dentistry & Public Health, Faculty of Dentistry, Sinai university, Kantara Campus, Ismailia, Egypt

**Keywords:** endodontics, sodium hypochlorite, temperature

## Abstract

**Objective:**

The aim of this study was to evaluate the temperature changes of sodium hypochlorite (NaOCl) after the use of different activation techniques: passive ultrasonic irrigation (PUI), XP-Endo Finisher (XP), and Easy Clean (EC).

**Materials and Methods:**

Thirty patients were selected for this randomized crossover study and each patient received root canal treatment in maxillary incisor. Each tooth was subjected to three activation techniques in a random order in the final irrigation of the treatment. All irrigation was performed using a 2.5% NaOCl solution, with the solution's temperature stabilized at 21°C (baseline) in the syringe. No agitation was used as a control before the use of the devices. After each activation technique, the intracanal temperature was measured using a K-type thermocouple. Statistical analyses were performed using Kruskal–Wallis and Dunn tests.

**Results:**

Temperatures remain in the range of 21.0 to 21.2°C at baseline and were higher in the control (29.5–34.1°C), PUI (29.9–34.2°C), EC (29.8–35.6°C), and XP (29.9–34.7°C) groups. The temperature average of the baseline period was inferior to those observed in all groups; moreover, despite of temperature changes among the control and all experimental groups no difference between them were identified.

**Conclusion:**

The temperature increase caused by activation with PUI, EC, and XP was similar and did not exceed the levels observed when no agitation was performed.

## Introduction

Endodontic failure is often characterized by persistent symptoms and periapical lesions. Periapical lesion is a root canal biofilm-mediated disease characterized by the presence of proinflammatory cytokines responsible for stimulating osteoclastogenesis and bone resorption in the periapical area. 1 2 When a periapical lesion develops, canal disinfection is a critical process for the success of root canal therapy. However, due to the complexity of the root canal system, such as the presence of canal curvature, irregularities, ramifications, isthmuses, lateral canals, and apical deltas, 3 4 the use of mechanical instrumentation alone is not sufficient to achieve maximum disinfection and removal of necrotic debris from the root canal, especially in apical areas that are difficult to reach during mechanical instrumentation.

Irrigation plays an essential role to improve the root canal disinfection. 3 Sodium hypochlorite (NaOCl) solution is a well-known irrigant solution for root canal disinfection due to its antibacterial activity and ability to promote dissolution of organic matter. 5 6 Even though NaOCl is the most used irrigant solution, it cannot eliminate inorganic components from the dentine walls and debris formed during mechanical instrumentation. 7 8 Due to this, the use of higher concentrations and preheating of the irrigant have been proposed as methods to enhance its effectiveness 9 ; however, side effects from using high-concentration NaOCl, such as tissue damage and postoperative swelling, can occur. 5 9

Several studies have explored the heating of NaOCl as a strategy to enhance its efficacy. 9 Cunningham and Joseph 10 compared the tissue dissolution capacity of 2.6 and 5.2% NaOCl at temperatures of 21 and 37°C, finding that the 2.6% solution at 37°C was as effective as 5.2% NaOCl at 21°C. Similarly, Sirtes et al 11 observed that 1% NaOCl heated to 45°C had a superior tissue dissolution capacity compared with 5.25% NaOCl at 20°C, and a 100-fold increase in antimicrobial efficacy between solutions at 20 and 45°C. Despite these promising findings, the ideal temperature for each concentration of NaOCl has yet to be established to maximize its beneficial effects while minimizing associated risks.

Although preheating the NaOCl solution has been demonstrated to improve tissue-dissolving and antimicrobial effects, a previous *in vivo* study showed that its efficacy is limited due to the time in which the temperature is maintained when the solution is injected into the root canal. 11 Therefore, the use of intracanal irrigation using activation techniques is of great interest since they are already used as auxiliary procedures to optimize cleaning of infected root canals and have been suggested to increase NaOCl solution temperatures. 12 13 For example, Zeltner et al 12 demonstrated, *in vitro* , that passive ultrasonic irrigation (PUI) is an activation technique associated with an increase in NaOCl solution temperature.

PUI is a commonly suggested activation protocol that uses the acoustic transmission of irrigant as a substitute for traditional irrigation methods. Although the efficacy of PUI is reported in the literature, new methods have been developed to achieve improved results, especially regarding increased penetration of the irrigant in the apical third of the root canal, including rotatory instruments such as the XP-Endo Finisher (XP) and Easy Clean (EC). 13 14

There are no *in vivo* studies that assess the temperature increase following different activation techniques. Furthermore, existing studies are limited to *in vitro* evaluations and lack a control group without activation for comparison. This study aimed to assess the *in vivo* temperature variations of NaOCl solution when different activation techniques, including PUI, XP, and EC, were used for root canal irrigation. The null hypothesis proposed that there would be no significant difference in the intracanal NaOCl temperature increase among the three activation techniques and when no agitation is performed.

## Materials and Methods

This study was approved by the local Research and Ethics Committee of São Leopoldo Mandic Dental Research Center (protocol number 2.540.741). Moreover, this study is reported according to the Consolidated Standards of Reporting Trials Statement (CONSORT, 2010) and registered with the Brazilian Registry of Clinical Trials (REBEC: RBR-2CP8T8). An *a priori* sample size calculation was performed using G*Power software version 3.1 for Macintosh (Heinrich-Heine, Universität Düsseldorf, Düsseldorf, NRW, Germany) and the results of a previous study 15 with respect to the expected changes in temperature. The power calculation based on a type I error of 0.05 and a power of 95% suggested a minimum sample size of 30 samples in each group to detect differences between treatment protocols. All patients participating in the study were informed of the risks, benefits, and treatment protocol prior to taking part in the trial and signed the Free Agreement Formulary. The patients were enrolled from November 2017 to May 2018. Systemically healthy patients aged 19 to 58 years attending a private clinic, in need of and predisposed to undergo root canal treatment of the maxillary central or lateral incisors with a single canal, closed apices, and root canal curvature up to 5 degrees were included. The diagnosis was established according to the patient's history and standardized clinical and radiographical examinations. Patients were excluded from the study if they were not willing to participate or if they presented with severe systemic disease or contraindications for endodontic treatment.

**Fig. 1:**
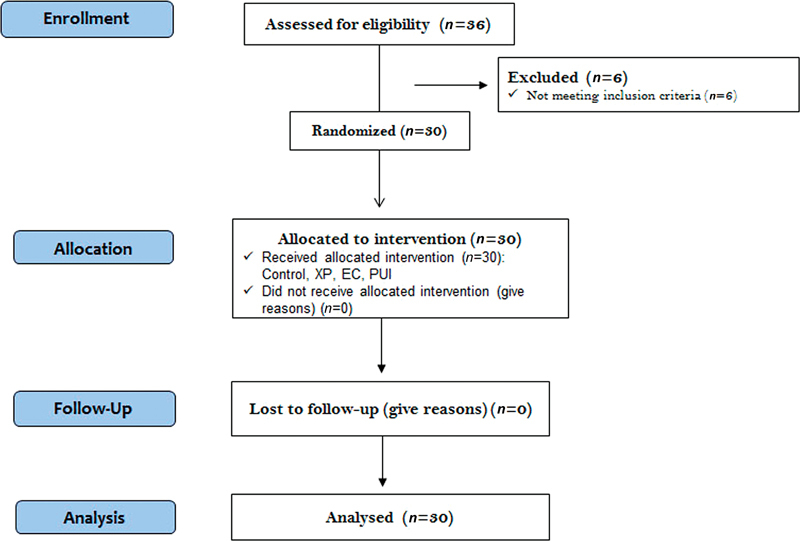
A Consolidated Standards of Reporting Trials Statement (CONSORT) flowchart of the participants throughout the trial.

Given the considerable diverse factors affecting temperature changes inside the root canal in human research subjects, this study was conducted with a crossover design to improve sensitivity for detection of temperature changes, as initial temperature of each subject would serve as his/her own control, thus results are not confounded by differences in health states. There were no changes to the study method after trial commencement. In total, 30 patients were selected for this crossover study in which each patient received root canal treatment of only one maxillary central or lateral incisor. All patients were allocated to receive a random sequence of interventions, where each patient underwent treatment with 2.5% NaOCl irrigation using three different irrigant agitation devices in a randomized order: (1) PUI, (2) EC, and (3) XP. Initially, the temperature inside the root canal of each patient was measured and recorded as a control (no agitation) before using the devices. The sequence of interventions was then performed for each patient, with each intervention period separated by a 180-second run-in period without agitation to allow for biological thermoregulation before starting the next irrigant agitation device. The primary outcome of this study was the temperature changes of NaOCl solution measured immediately after the use of different activation techniques. There was no follow-up phase. The flow diagram of the study design is presented in Fig. 1 .

## Root Canal Preparation

All clinical procedures, including the root canal treatments, were performed by a single operator (an endodontist) to minimize potential variability caused by human factors and to ensure the reliability and reproducibility of the results. A total of 30 maxillary central or lateral incisors were anesthetized with 2% mepivacaine with 1:100,000 noradrenaline (DFL, Rio de Janeiro, Brazil), isolated with a rubber dam (Maquira Indústria de Produtos Odontológicos, Maringá, Brazil) to protect teeth from saliva leakage. The operating field was disinfected with 30% hydrogen peroxide followed by 5% tincture of iodine, according to Möller's protocol. 13 Next, after access cavity preparation using a sterile diamond-coated spherical bur 1014 (KG Sorensen, Cotia, Brazil), the working length (WL) was estimated using the preoperative radiograph. Apical patency was then verified with a size 15 K-file (Dentsply Sirona Endodontics, Ballaigues, Switzerland), and the WL was established using an electronic apex locator (EAL) Raypex 6 (VDW, Munich, Germany) set 1.0 mm shorter than the distance measured using an EAL and radiographically confirmed. Finally, root canals were prepared using a WaveOne Gold medium single file (35.06) (Dentsply Maillefer, Ballaigues, Switzerland) in a reciprocating motion driven by an endodontic electric motor (VDW), according to the manufacturer's recommendations. After each cycle, the canals were irrigated with 5 mL of 2.5% NaOCl solution (Asfer Ind. Química Ltda., São Caetano do Sul, Brazil) using a Monoject syringe with a 25-gauge needle placed 2 mm short of the WL. Once the root canal preparation was completed, the root canal was dried using paper points after the final irrigation (Tanari, Manacapuru, Brazil).

Additional irrigation was performed using a 2.5% NaOCl solution, with the solution's temperature stabilized at 21°C (baseline) in the syringe. Before intracanal insertion, the temperature of the irrigation solution was measured and maintained at 21°C by storing it in a container submerged in thermostatically controlled water. After irrigation, the canals were dried with sterile absorbent paper points, and a 180-second waiting period was observed before reintroducing the 2.5% NaOCl solution for each test group. The temperature was recorded before the canal was irrigated again, dried, and another 180-second interval was observed before starting the next group.

In the control group, which represented conventional irrigation without agitation, the temperature was only recorded. Following this, the same tooth underwent a sequence of three activation techniques for the final irrigation of the root canal. The sequence for each patient was determined using blocked randomization (block size = 5) via the Web site www.random.org . Each tooth was subjected to the three agitation techniques and the control condition, forming four distinct groups. The sequence of technique application was determined by randomly drawing lots from the 24 possible combinations. This number of combinations is derived from the permutation of the four techniques, ensuring that all possible application orders were considered. Due to the study's methodology, only the outcome assessors were blinded. The activation techniques were performed according to the manufacturers' instructions and were performed as follows.

### Passive Ultrasonic Irrigation

Ultrasonic irrigation was performed by activating the 2.5% NaOCl solution in the canals using the Irrisonic 20.01 stainless steel instrument (Helse Dental Technology, Santa Rosa de Viterbo, Brazil) coupled to an ultrasound unit (Gnatus, Ribeirão Preto, Brazil). The instrument was placed 1 mm from the WL 14 and was activated at a low frequency (3 kHz) for 20 seconds.

### Easy Clean

The 2.5% NaOCl solution was activated using the EC instrument, size 25/04 (Easy Dental Equipment, Belo Horizonte, Brazil), in continuous rotation coupled to a counter-angle connected to a micromotor with an operating speed of approximately 20,000 rotations per minute (rpm) (KaVo Kerr Group, Charlotte, United States). 8 The EC instrument was inserted 1 mm short of the WL, and the activation was performed for 20 seconds.

### XP-Endo Finisher

The activation of 2.5% NaOCl solution was performed using the XP (FKG Dentaire, La Chaux-de-Fonds, Switzerland) coupled to an endodontic motor X-Smart Plus (Dentsply Maillefer) with lengthwise movements up to 1 mm from the WL, at a speed of 800 rpm and a torque of 1 Ncm 12 for 20 seconds.

## Intracanal Temperature Measurement

The intracanal temperature was measured using a sterile K-type thermocouple (0.2 mm in diameter) (Omega Engineering Inc, Stamford, United States) connected to a 0.1°C precision digital thermometer (Salvi Casagrande Ltda, São Paulo, Brazil) previously calibrated by the Brazilian Network of Calibration (RBC). After performing each activation technique, the K-type thermocouple probe was placed 1 mm short of the WL to measure the intracanal temperature. The insertion depths were verified using silicone stops. The measuring probe was positioned in the root canal for a period of 3 seconds to record the temperature. The highest temperature ( *T*_max_ ) and fold change (difference in temperature between baseline and *T*_max_ ) were established as the average of the temperature recorded for the four groups in all 30 samples. Since the NaOCl solution was at 21°C initially, any rise in temperature was ascribed to the activation technique.

## Root Canal Filling

After the temperature measurements, all teeth were irrigated with 5 mL of 17% ethylenediaminetetraacetic acid (Biodinâmica Quím e Farm Ltda, Ibiporã, Brazil) for 3 minutes followed by irrigation with distilled water following previous investigations. 16 The canal was dried with paper points and filled using a size 30.06 gutta-percha cones (Tanari, Manacapuru, Brazil) with AH Plus sealer (Dentsply Maillefer), according to Tagger's hybrid technique. Finally, the tooth was restored with Filtek Z350 (3M ESPE, Sumaré, Brazil) composite resin.

## Statistical Analyses

Data were evaluated with GraphPad Prism version 5.0.0 for Macintosh (GraphPad Software, San Diego, United States). The Shapiro–Wilk method was adopted to verify the normality of data. As the samples did not have a normal distribution, differences in intracanal temperature measurements between groups were assessed using Friedman's test ( *p* < 0.05).

## Results

**Fig. 2:**
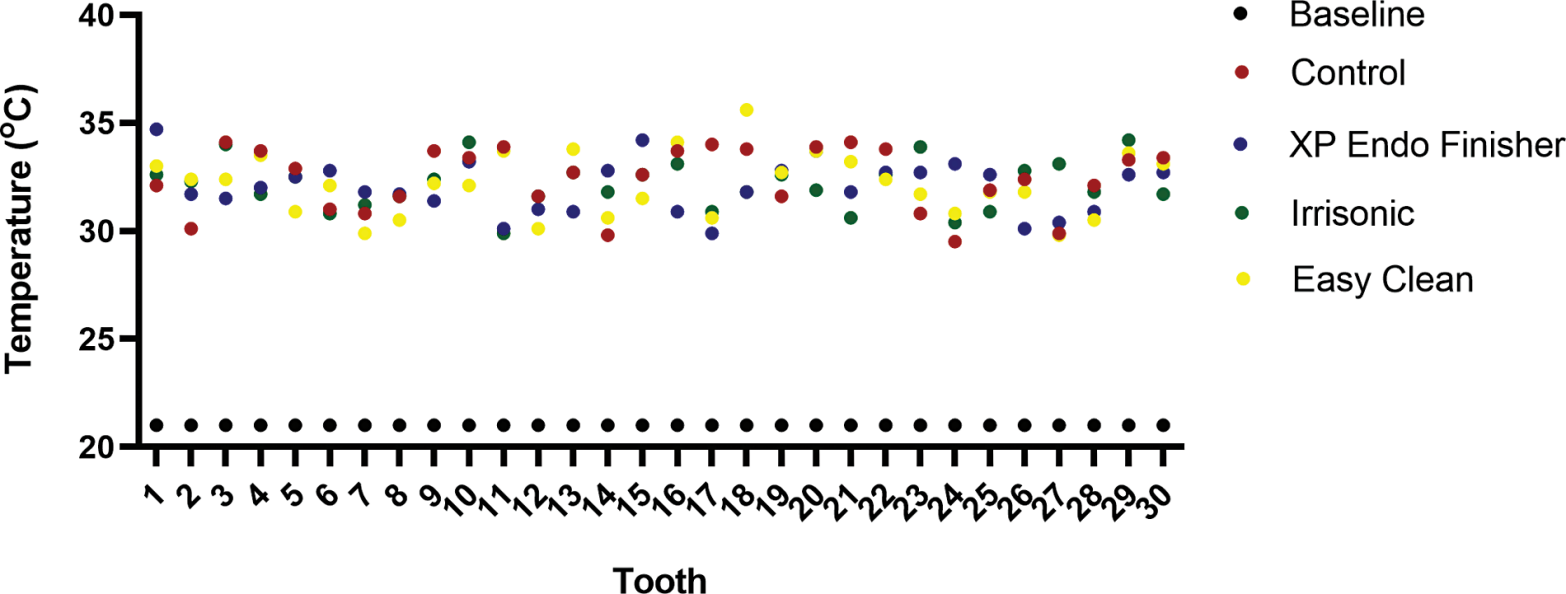
Temperature (°C) recorded at the apical levels of root canals in all samples (tooth) before (baseline) and after irrigation with the use or not (control) of passive ultrasonic irrigation (PUI), Easy Clean (EC), and XP-Endo Finisher (XP).

Characteristics of the study population are shown in Table 1 . The average age of the subjects was 32.57 ± 11.27 years. In terms of gender, 40% was male and 60% was female. Regarding tooth, the distribution of maxillary central incisor and maxillary lateral incisor for the root canal treatment was 56.7 and 43.3%, respectively. Out of 36 patients enrolled in the study, 06 patients were excluded according to the study criteria; therefore, 30 patients were included in the final analysis ( Fig. 1 ). Fig. 2 summarizes the overall results of 120 temperature measurements obtained from all activation techniques groups performed in the root canal treatment.

**Table 1:** Baseline demographic and clinical characteristics of the study population

Characteristic	
Demographic data	Study group ( *n* = 30)
Age (y)	32.57 ± 11.27
Male ( *n* , %)	12 (40.0)
Female ( *n* , %)	18 (60.0)
Tooth	( *n* , %)
Maxillary central incisor	17 (56.7)
Maxillary lateral incisor	13 (43.3)

Note: Age (years) is reported as mean values ± standard deviation.

**Fig. 3:**
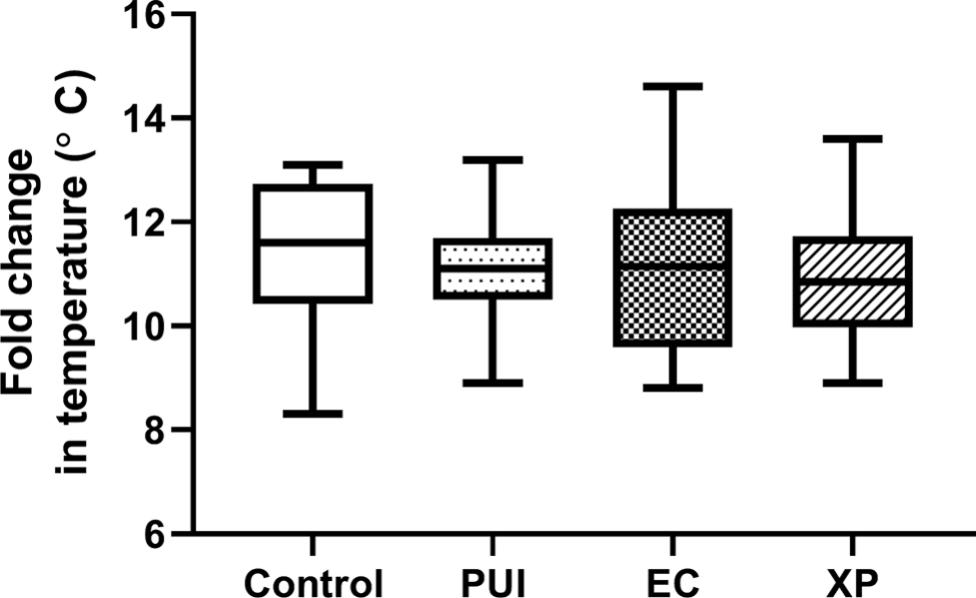
Box-plot of fold changes in temperature (°C) for the control, passive ultrasonic irrigation (PUI), Easy Clean (EC), and XP-Endo Finisher (XP) groups demonstrate the difference in temperature between baseline (initial period) and temperature recorded after the use or not of activation techniques.

The baseline period exhibited a lower and more stable temperature due to the equilibrium established before 180 seconds of final irrigation to standardize all groups with the same conditions. Temperatures were stable at 21.0 to 21.2°C in the baseline period, whereas variations were detected in the control (29.5–34.1°C), PUI (29.9–34.2°C), EC (29.8–35.6°C), and XP (29.9–34.7°C) groups. The temperature average of the baseline period was inferior to those observed in all groups ( *p* < 0.0001) showing that the raise of temperature occurred independent of activation ( Table 2 ). On the other hand, temperature changes were noted in the control and all experimental groups ( Table 2 ); moreover, these changes observed in control group were similar to all experimental groups ( *p* = 0.6336) ( Fig. 3 ).

**Table 2:** Intracanal temperature ( *T*_max_ ; °C) recorded before (baseline) and after irrigation with the use of different activation techniques

Group	*N*	Mean	SD	Median	Minimum	Maximum
Initial (baseline)	30	21.03	0.05	21.00 ^b^	21.00	21.20
No activation (control)	30	32.41	1.45	32.65 ^a^	29.50	34.10
Passive ultrasonic irrigation	30	32.14	1.11	32.10 ^a^	29.90	34.20
Easy Clean	30	32.14	1.43	32.15 ^a^	29.80	35.60
XP-Endo Finisher	30	32.03	1.20	31.90 ^a^	29.90	34.70

Abbreviation: SD, standard deviation.

Note: Different superscript lowercase letters indicate significant differences between groups ( *p* < 0.05; Dunn test).

## Discussion

Overall, the results of this study demonstrate an increase in NaOCl solution temperature inside the root canal independent of the activation method during the irrigation process. The use of PUI, EC, or XP was not enough to clinically increase the intracanal temperature of NaOCl relative to the results found when no agitation was performed; these results support the null hypothesis.

Heating NaOCl has an important role in improving of tissue-dissolving effects. 17 The use of mechanical activation has already been suggested to increase the temperature inside the root canal, but studies regarding irrigant activation are limited to the PUI technique. However, there is currently no consensus regarding the temperature increase recorded at the apical levels of root canals after PUI, with reported results varying from a mean elevation of 0.6 18 to 4.2°C, 12 while the temperature rise observed in this study was 11°C. In fact, it is important to highlight that the controversial results are possibly due to the differences in methodology. In previous studies, the authors used different ultrasonic devices and human canines for *in vitro* evaluation, while the present study used the maxillary central or lateral incisor and considered the clinical situation.

Considering the diverse factors affecting temperature changes inside the root canal in a clinical situation, such as the water content of hard and soft tissues, presence of dentinal tubules, and the heat sink capacity of the periradicular vascular system, 11 19 it can be assumed that the use of *in vivo* intracanal temperature measurements seems to be a more reliable methodology to translate the results to a clinical scenario. In this study, maxillary incisors were selected because the root canal anatomy provides additional facilities to control the variability between the groups, such as the presence of large diameter canals, which are related to the highest temperature changes. 19 Furthermore, the temperature measurements were done in the apical part of the root canal because it is the most difficult area to be effectively irrigated 11 20 and the interval of 180 seconds between agitation techniques was established to achieve biological thermoregulation.

It is interesting to note that the control group, which presented the lowest maximum temperature, had temperature increases similar to that following all activation techniques evaluated ( *p* = 0.6336), and even with no agitation, there was still a rise in temperature from that at baseline. Moreover, the maximum temperature achieved was approximately 35°C, which is close to the average normal body temperature (37°C). 10 21 These results highlight the direct relationship between the thermoregulation system of humans and intracanal temperature changes, independent of the use of NaOCl agitation. The finding is in concordance with that of Cunningham and Joseph 10 who found that the equilibrium of NaOCl solution at room temperature inside the mandibular incisor and maxillary canine, premolar, and first molar was within the range of 31 to 33.5°C after 1 to 2 minutes. An *in vivo* study by de Hemptinne et al 11 also showed rapid temperature stabilization at 35.1°C in the apical part of the root canal, even when the NaOCl was preheated to 66°C in the syringe before injection. Thus, since the factors related to temperature changes were standardized in this study using only maxillary incisors and the same tooth for comparison between groups (e.g., root canal diameter, thickness, and tooth size), the temperature data after activation or nonactivation of the irrigant solution might be linked to thermoregulatory mechanisms. The body's natural thermoregulation maintains an internal temperature within a narrow range of approximately 36.5°C. This thermoregulation is primarily mediated by the vascularization of the periodontium, particularly the periodontal ligament, which is highly vascularized and surrounds both the root portion of the tooth and the inner wall of the alveolar bone. 22 The periradicular vascular system plays a role in dissipating heat and stabilizing body temperature through blood circulation and the thermal conductivity of the periodontal membrane, radicular dentin tubules, and alveolar bone. 11 19 22 23 Additionally, the small volume of the irrigating solution can lead to rapid temperature changes, facilitating heat transfer from the periodontal tissue to the solution. This thermal conduction could explain the observed temperature increase in all groups, including the control, and provides an understanding of the results.

Moreover, this is the first study comparing different activation systems in an *in vivo* situation. Most previous studies did not consider recent instruments, such as the EC and XP, 10 11 13 18 which seem to have good results in improving root canal cleaning. 13 24 Overall, the findings and methodology in this study can help elucidate if the use of activation techniques aiming to increase NaOCl temperature significantly contributes to the effectiveness of root canal irrigation. However, although a strength of this study includes a crossover design, the generalizability of the findings should be applied with caution, since patients with systemic disease were not evaluated, and due to the use of different activation techniques, the blinding of the operators to the experimental groups was not possible, addressing a source of potential bias. In addition, among the limitations of the study is that it represents the best-case scenario as done in maxillary incisors with wide canals. Future studies should evaluate the relationship between temperature and antimicrobial and tissue-dissolving activity *in vivo* to better understand the thermodynamic mechanisms of irrigant solutions inside root canals.

## Conclusion

Thus, it can be concluded that the temperature increase caused by activation with PUI, EC, and XP was similar and did not exceed the levels observed when no agitation was performed.
